# Seasonality of COVID-19 incidence in the United States

**DOI:** 10.3389/fpubh.2023.1298593

**Published:** 2023-12-05

**Authors:** El Hussain Shamsa, Ali Shamsa, Kezhong Zhang

**Affiliations:** ^1^Center for Molecular Medicine & Genetics, Detroit, MI, United States; ^2^Department of Internal Medicine, University Hospitals Cleveland Medical Center, Case Western Reserve University, Cleveland, OH, United States; ^3^Medical College of Wisconsin, Milwaukee, WI, United States; ^4^Department of Biochemsitry, Microbiology, and Immunology, Wayne State University School of Medicine, Detroit, MI, United States

**Keywords:** COVID-19, seasonality, public health, infectious diseases, periodicity

## Abstract

**Background:**

The surges of Coronavirus Disease 2019 (COVID-19) appeared to follow a repeating pattern of COVID-19 outbreaks regardless of social distancing, mask mandates, and vaccination campaigns.

**Objectives:**

This study aimed to investigate the seasonality of COVID-19 incidence in the United States of America (USA), and to delineate the dominant frequencies of the periodic patterns of the disease.

**Methods:**

We characterized periodicity in COVID-19 incidences over the first three full seasonal years (March 2020 to March 2023) of the COVID-19 pandemic in the USA. We utilized a spectral analysis approach to find the naturally occurring dominant frequencies of oscillation in the incidence data using a Fast Fourier Transform (FFT) algorithm.

**Results:**

Our study revealed four dominant peaks in the periodogram: the two most dominant peaks show a period of oscillation of 366 days and 146.4 days, while two smaller peaks indicate periods of 183 days and 122 days. The period of 366 days indicates that there is a single COVID-19 outbreak that occurs approximately once every year, which correlates with the dominant outbreak in the early/mid-winter months. The period of 146.4 days indicates approximately 3 peaks per year and matches well with each of the 3 annual outbreaks per year.

**Conclusion:**

Our study revealed the predictable seasonality of COVID-19 outbreaks, which will guide public health preventative efforts to control future outbreaks. However, the methods used in this study cannot predict the amplitudes of the incidences in each outbreak: a multifactorial problem that involves complex environmental, social, and viral strain variables.

## Introduction

Infection with SARS-CoV-2, the virus which caused the world-wide Coronavirus Disease 2019 (COVID-19) pandemic, was first recorded in late 2019. The three continents with the highest burden of disease are North America, Europe, and Asia (1). The United States (US) has the highest case burden and has recorded approximately 1 million deaths (2).

It has been debated whether COVID-19 disease incidence follows a seasonal pattern like many other respiratory viral infections, such as influenza, rhinovirus, enterovirus, human parainfluenza (HPIV), respiratory syncytial virus (RSV) (Supplementary Table S1) (3–5). The identification of seasonality of these viruses has been crucial for infection control as it has guided public health prevention measures, such as serving as the basis behind the annual Fall influenza vaccine prior to the yearly winter influenza epidemic. As such, we expect that seasonality studies will be valuable in combating the COVID-19 pandemic. Seasonality, in terms of a disease, can be defined in either a literal manner where disease incidence peaks correlate with a particular period of the calendar year or, alternatively, where incidence peaks and troughs recur at regular intervals, representing a periodic sinusoidal process (6). Though, at first, scientists associated the surges of COVID-19 with holidays, lack of social distancing, and mask mandates, there appears to be a repeating pattern of COVID-19 outbreaks regardless of whether public health measures are in place (e.g., social distancing measures, mask mandates, and vaccinations). The probability of COVID-19 seasonality gained momentum as, in many Western countries, the COVID-19 incidence receded during the Summer months, climbed back up in the Fall and reached its peak in the Winter during the pandemic. Seasonal spikes in COVID-19 cases have been studied in multiple countries and the existence of patterns in disease surges has been identified (2, 7–9), however literature characterizing regular, predictable seasonal oscillations of cases over multiple years of data in the United States is lacking.

Identification of the seasonality of COVID-19 is an important step towards elucidating the causes of disease surges and will also offer possibilities for increased disease preparedness and preventive strategies. This is crucial for the development of more effective and targeted public health policies to reduce viral transmission, such as vaccine timing, COVID-19 screening initiations, and nonpharmacological interventions. The effectiveness of COVID-19 vaccines has been found to peak approximately 1–2 months after administration and thereafter gradually decline over time (10). Thus, predicting COVID-19 incidence surges is essential in guiding vaccination timing so that peak vaccine effectiveness can be aligned with predicted case spikes. SARS-COV-2 viral screening of symptomatic patients, especially for those who are in high risk settings, and their direct contacts has also been found to be effective in reducing viral transmission (11, 12), and by identifying case surge timings officials can screen high-risk populations during peak seasons. Lastly, increased public awareness on effective social strategies, such as face masks, hand washing, and social distancing, can be encouraged to reduce transmission during peak seasons (13).

In our study, we analyzed the trajectory of the daily confirmed cases of COVID-19 in the US using a spectral analysis approach to determine whether there exists seasonality in the occurrence of the disease. Spectral analysis is based on the idea that any waveform can be represented as a sum of sine waves at different frequencies with different phase relationships and amplitudes; it is a method which can be used to calculate dominant frequencies of oscillation in a set of sequential data (14, 15). Our analyses focused on the first two full seasonal years of the COVID-19 pandemic, spanning from Spring 2020 to Spring 2022, when the COVID-19 pandemic was in full swing and when the daily incidence data reporting was most accurate. The results indicate that there exists a regular periodicity in COVID-19 incidences with multiple predictable peaks and troughs each year.

## Resources and methods

### Dataset collection

Our study analyzed daily, county-wise data spanning two full seasonal years (March 19, 2020, to March 20, 2022) for the contiguous United States. Case data was downloaded from the 1Point3Acres COVID-19 database.^1^ This database contains real-time updated, confirmed COVID-19 cases and deaths throughout the US and is used by Johns Hopkins University in their global COVID-19 tracking project, the US Center for Disease Control (CDC), and other renowned institutions. We also obtained data from the 2022–2023 (spanning from March 21, 2022, to March 23, 2023) by combining data from the 1Point3Acres database (final day of case reporting was February 13, 2023) with the New York Times database (final day of case reporting was March 23, 2023), which is shown in Supplementary Figure S1. Though there exist multiple COVID-19 datasets available to the public, we chose the 1Point3Acres and New York Times datasets due to their reliable, transparent county-level data collection procedures. The cross-validation steps used in making their datasets are clear, rigorous, and publicly available, unlike the CDC and Johns Hopkins University COVID trackers, whose data aggregation procedures are not entirely transparent (16, 17).

The 2022–2023 seasonal year was not included in the seasonality analyses, as testing and case reliability were suboptimal in the final year of case tracking, resulting in the significant noise in the data seen in Supplementary Figure S1. Although this noisiness is clear by observation of the raw data in Supplementary Figure S1, it becomes even more clear when comparing the mean COVID-19 incidence of 62,547 daily cases for the 2022–2023 year with a standard deviation larger than the mean at 62,582. We can further illustrate this by separating the data into portions defined by seasons: the Spring season had an average of 67,396 daily cases and standard deviation of 61,472 cases, the Summer season had an average of 98,248 daily cases and standard deviation of 77,255 cases, the Fall season had an average of 40,464 daily cases and standard deviation of 41,867 cases, and the Winter season had an average of 42,087 daily cases and standard deviation of 43,717 cases. The fact that the standard deviation is similar to, and at times greater than, the mean in each portion of the dataset provides a rough quantitative measure of the extensive noisiness of the data. For this reason, any quantitative conclusions of seasonality using this final seasonal year of data would be unreliable, and thus we use this data for observational and anecdotal purposes only.

### Spectral analysis

The daily national case data obtained from 3/19/2020 to 3/20/2022 (*n* = 732 days), was used to construct a time series with daily COVID-19 cases as the value at each individual timepoint, which in our study are days (18). In the following spectral analyses, we use centered, log-transformed data, which was obtained from the raw time series as shown in Eq. 1:


(1) $$x_{t}=\ln\left(r_{t}\right)–\ln\left(\frac{\sum_{t=1}^{n}r_{t}}{n}\right)$$

**Figure 1:**
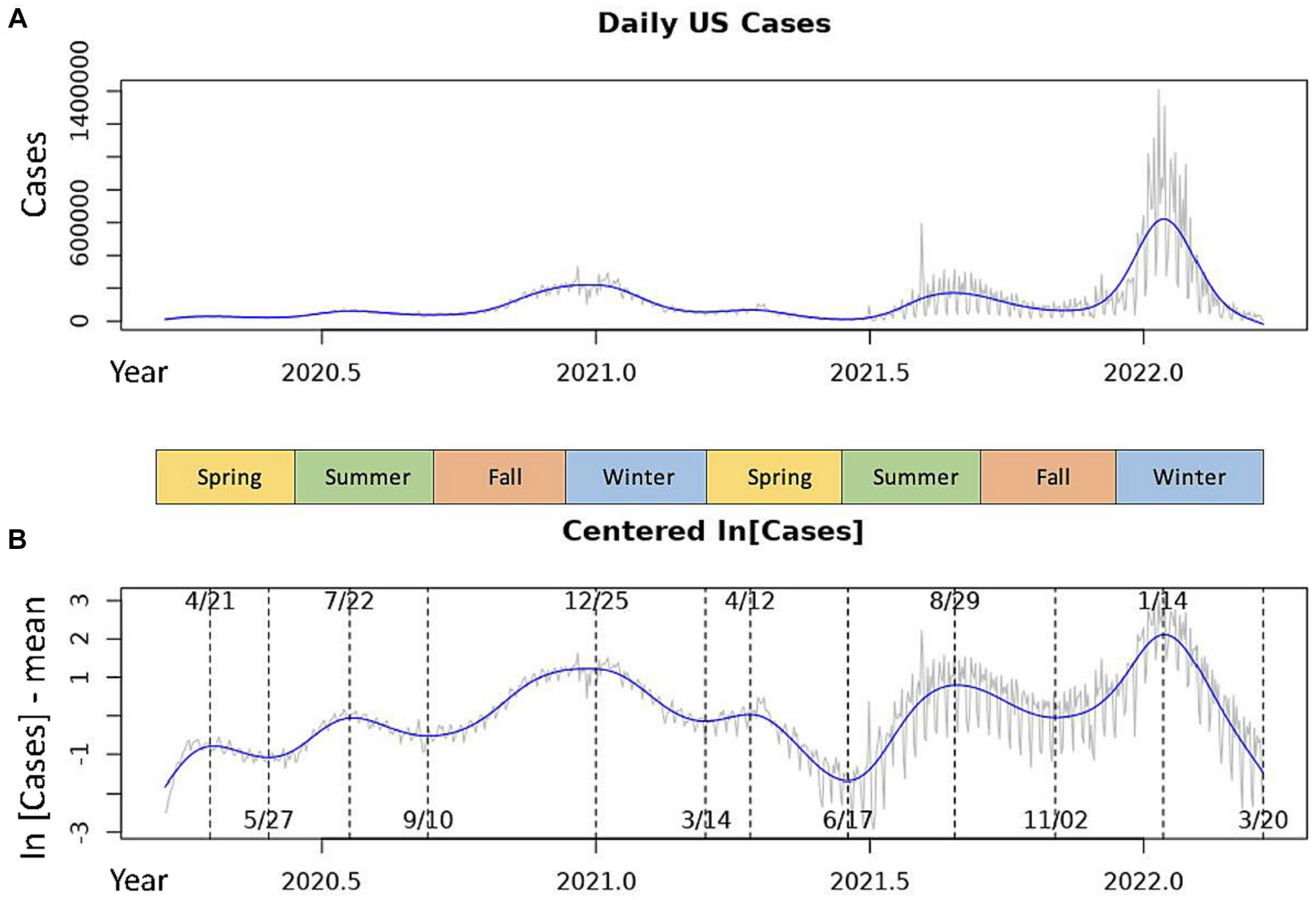
Daily COVID-19 incidences in the USA over the 2020–2022 seasonal years. The gray line represents the daily measures for the raw data time series. The blue line is the smoothed cubic spline fit to the time series. **(A)** Shows the raw national daily case data. **(B)** Shows centered, log-transformed daily cases with inflection points of the spline marked by dotted lines. Dates of local maxima are shown at the top of the graph, those of local minima are shown at the bottom.

Where *x_t_* is the centered time series, *r_t_* is the time series which represents the raw data, and *t* = 1, …, *n* represents each time point where *n* = 732 days. Log-transformation of the raw time series data was preferred due to the significant variations in the amplitudes of the case spikes as the time series progresses. The log-scale allows for graphical visualization and comparison of all peaks despite these large variations in amplitude. Centering the data allows for easier identification of distinct peaks and troughs by allowing for pleasant visualization of each with respect to the mean of the time series. A cubic smoothing spline was fit to this data by fitting the cubic polynomials *x_t_* = *m_x,t_* + *w_x,t_* and *r_t_* = *m_r,t_* + *w_r,t_* where:


(2) $$m_{t}=\beta_{0}+\beta_{1}t+\beta_{2}t^{2}+\beta_{3}t^{3}$$

and *w_t_* represents random noise. Each *m_t_* is calculated by regressing over *n* intervals, then a smoothing parameter is used to determine an appropriate degree of smoothness while avoiding over-smoothing. These cubic smoothing spline procedures were conducted using a spline function in R. The resulting smoothed splines are shown as the blue line in Figure 1.

Spectral analysis is an approach to time series analysis in which periodic components of a time series are assumed to follow a sinusoidal oscillation (18). A periodogram was computed using a Fast Fourier Transform (FFT) algorithm in order to identify the predominant oscillatory frequencies, and thus periods, of the time series. Specifically, the periodogram, *I*(*j*/*n*), is calculated to identify the predominant oscillatory periods of a time series. Here, we define a period as the number of days (timepoints) for one cycle of oscillation. Thus, where frequency (⍵) of the oscillation is the number of cycles per day, the period of the oscillation is the inverse of frequency (period = 1/⍵). The periodogram is computed as follows:


(3) $$I\left(\frac{j}{n}\right)={\left|d\left(\frac{j}{n}\right)\right|}^{2}=\frac{1}{n}{\left(\overset{n}{\underset{t=1}{\sum }}x_{t}\cos\left(2\pi t\frac{j}{n}\right)\right)}^{2}+\frac{1}{n}{\left(\overset{n}{\underset{t=1}{\sum }}x_{t}\sin\left(2\pi t\frac{j}{n}\right)\right)}^{2}$$

For all *j* = 1, …, *n*/2 − 1. Here, *d*(*j*/*n*) is the Discrete Fourier transform (DFT) with Fourier (fundamental) frequencies ⍵_j_ = *j*/*n*. We then compute the DFT for each j using an FFT algorithm in R. The values *I* are thus computed for all frequencies between 0 and 0.5 and each of these values can be plotted to visualize the periodogram. The frequencies (⍵_j_) at which the value of *I*(⍵_j_) is largest represent the predominant frequencies of the time series, and the respective 1/⍵_j_ represent the dominant periods of oscillation. The scaled periodogram *P* can be calculated from *I* as follows:


(4) $$P\left(\omega_{j}\right)=\left(\frac{4}{n}\right)I\left(\omega_{j}\right)$$

**Figure 2:**
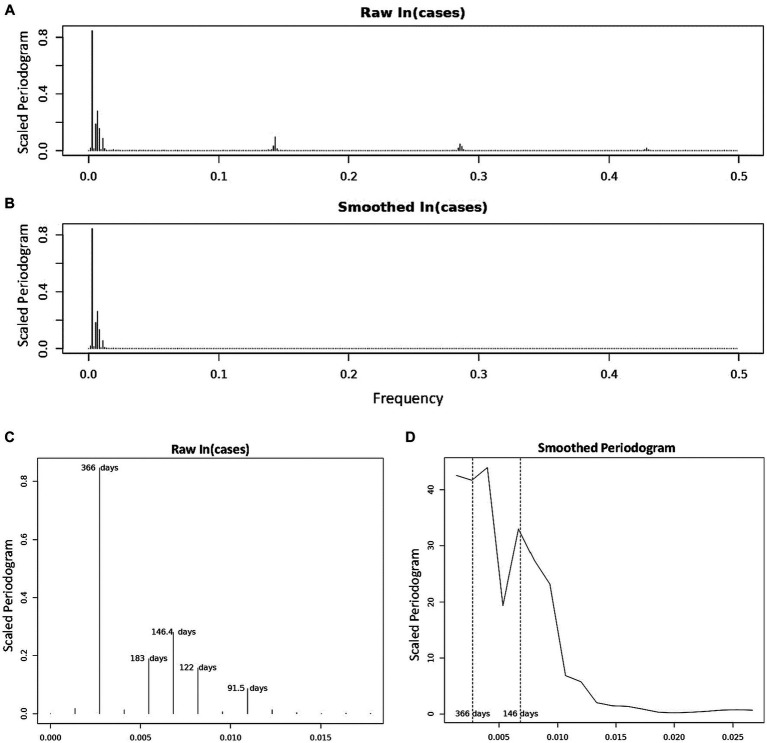
Periodogram of COVID-19 Incidence, where *n* = 732 days and frequency are number of cycles per 732 days (2 years). **(A)** Is the periodogram computed using the centered, log-transformed incidence data. **(B)** Is the periodogram computed using the values of the cubic spline obtained in Figure 1B. **(C)** Shows and labels the periods of the dominant peaks from **(A)**. **(D)** Is the smoothed periodogram computed using a Daniel kernel, as shown in Eq. 8.

Where the value of the scaled periodogram *P*(⍵_j_) at each *j* is the squared correlation that the sinusoid oscillating with frequency ⍵_j_ has with the sample data *x_t_*. For this reason, we used the values of the scaled periodogram in our analyses and in Figure 2 as it provides a much more intuitive measure of the periodogram values.

In order to visualize each of these dominant oscillatory frequencies identified using the periodogram calculations above, we can fit the theoretical sinusoids which oscillate at these frequencies to the COVID-19 incidence data. We will do this using multiple linear regression. Let:


(5) $$x_{t}=A\cos\left(2\pi \omega t+\phi \right)+w_{t}$$

Where $x_{t}$ is the centered, log-transformed incidence data as above, *A* is the amplitude of oscillation, *ɸ* is the phase shift, and *w_t_* is random noise. We can use the trigonometric identity:


(6) cos(a±b)=cos(a)cos(b)∓sin(a)sin(b)

to further expand Eq. 5, as follows:


(7) $$x_{t}=\beta_{1}\cos\left(2\pi \omega t\right)+\beta_{2}\sin\left(2\pi \omega t\right)+w_{t}$$

**Figure 3:**
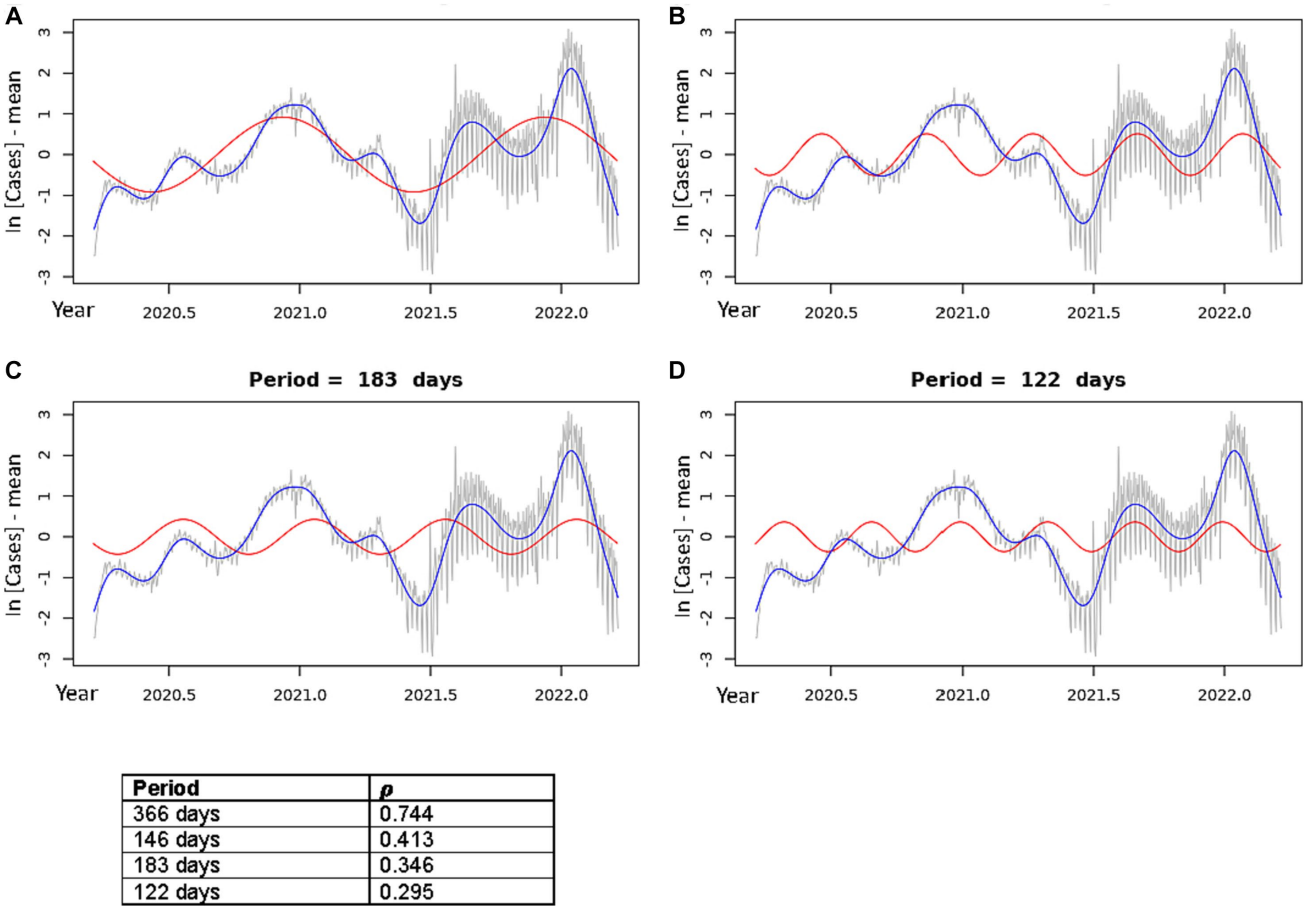
Best fit theoretical sinusoids shown with frequencies matching each of the four dominant peaks of the periodogram. Pearson’s correlation coefficient (𝝆) is shown for each plot (correlation of the sinusoid with the cubic spline). The gray line is the centered, log-transformed daily COVID-19 case data, the blue line is the cubic spline fitted to the data, the red line is the best fit theoretical sinusoid. **(A)** Shows the sinusoid with a period of 366 days, **(B)** shows the sinusoid with a period of 146.4 days, **(C)** shows the sinusoid with a period of 183 days, **(D)** shows the sinusoid with a period of 122 days.

**Figure 4:**
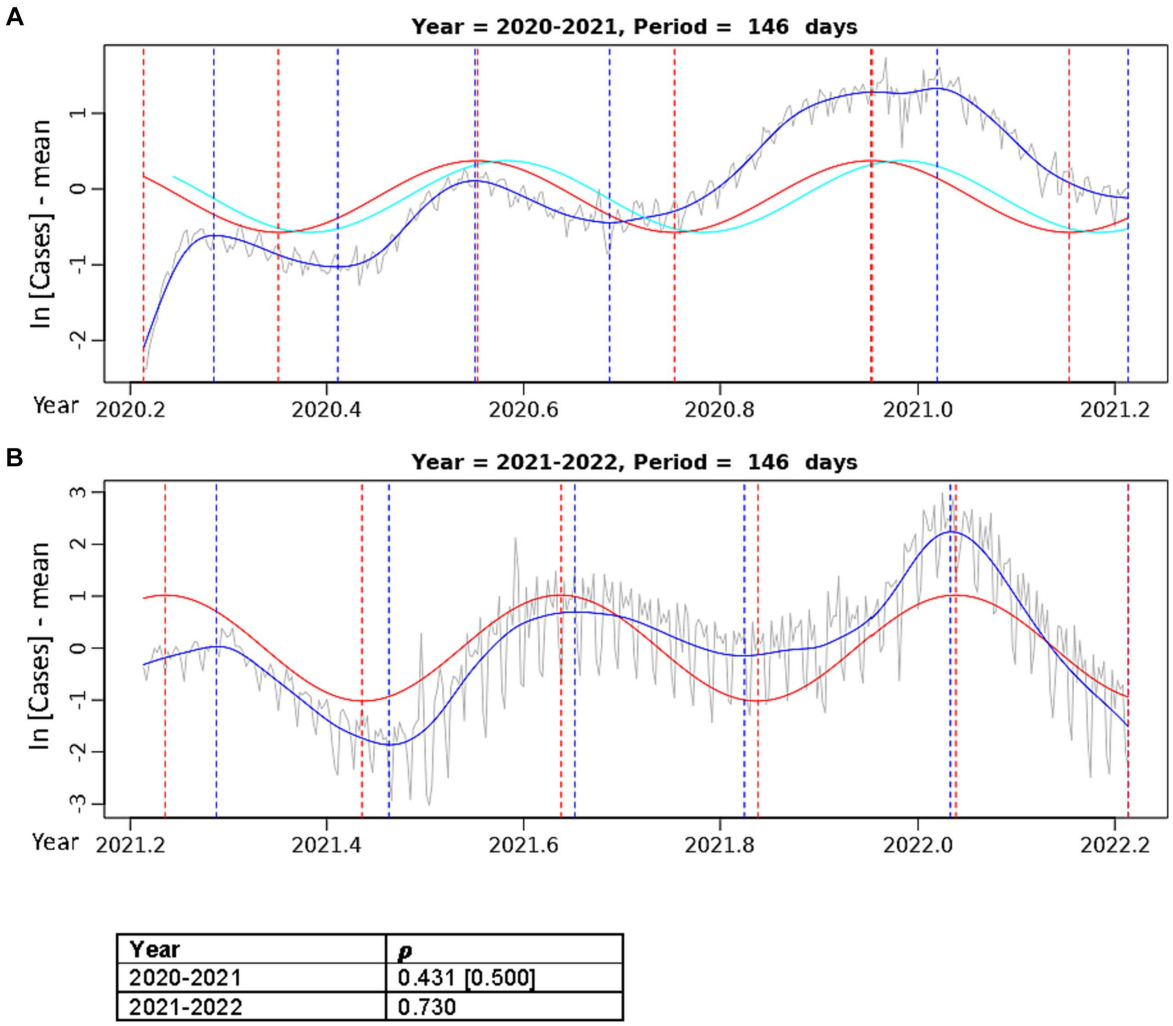
Best fit theoretical sinusoids with period 146.4 days fit to annual data. Pearson’s correlation (𝝆) of the sinusoid with the cubic spline is shown. The gray line is the centered, log-transformed daily COVID-19 case data, the blue line is the cubic spline fitted to the data, the red line is the best fit theoretical sinusoid. **(A)** Is the sinusoid fit to the data from the 2020–2021 seasonal year. The cyan line is the positive lagged (by 11 days) sinusoid. The Pearson’s correlation coefficient in the brackets is that of the lagged sinusoid with the cubic spline. **(B)** Is the sinusoid fit to the data from the 2021–2022 seasonal year.

Where *β*_1_ = *A* cos(*ɸ*) and *β*_2_ = −*A* sin(*ɸ*). By setting ⍵ = ⍵_j_ for each of the predominant Fourier frequencies identified in the parallelogram calculations, we obtain a linear equation for each frequency ⍵_j_ with outcome variable *x_t_*, regression coefficients *β*_1_ and *β*_2_, and explanatory variable *t*. By separating the phase shift term within the cosine expression (Eq. 5) using the above identities (Eq. 6), we can find the sinusoidal curve which best fits our time series data by using linear regression. When matched with the data, these best fit sinusoidal curves provide a clear illustration of the oscillations of the data and can be used to predict distinct peaks and troughs within the data (Figures 3, 4).

### Correlation of the series

Correlation of the theoretical best fit sinusoids with the data was tested by calculating Pearson’s 𝝆 for each sinusoid with the cubic spline fitted to the centered, log-transformed data. Furthermore, we confirmed that each sinusoid was in fact the best fit to the data by plotting cross-correlation functions (CCF, Supplementary Figures S3, S4), which show the [Pearson’s] correlation between the two-time series across various lags of the theoretical sinusoid.

### Statistical analyses

All statistical analyses detailed above were conducted in R (18, 19). All plots, tables, and figures were generated in both R and Microsoft Office Excel.

### Sensitivity analyses

We repeated these analyses using percent test positivity data to account for possible differences in testing habits over the study period and found no significant difference in results.

## Results

### Visualization of the COVID-19 case data

We observed dominant COVID-19 outbreaks that recur each year (Figure 1A). There exist smaller peaks in incidence that are more difficult to visualize in the raw data due to large variations in the amplitudes of case spikes. However, each of these peaks as well as the inflection points of the data more easily detectable with the centered, log-transformed data (Figure 1B). The annual periodicity of COVID-19 incidences appears to follow a pattern with 3 peaks each year, with one dominant peak in the Winter and two smaller peaks in early/mid-Spring and mid/late-Summer, and a significant drop in cases between each peak (Figure 1B). Using the cubic spline fitted to the data, we estimated the dates and case counts of each peak (Table 1). It should be noted that the estimated case counts at some of the peaks may not be accurate due to the significant amount of noise in daily cases seen around the peaks. Specifically, daily cases at the Summer and Winter peaks in 2022 reached much higher values in the raw data than estimated by the cubic spline. Thus, we include in Table 1 the maximum case counts from the raw data nearby each of these peaks. Though not included in the analyses, we observe that the above noted pattern of seasonal COVID-19 outbreaks was repeated in the 2022–2023 seasonal year, with incidence peaks in the Spring, Summer, and Winter (Supplementary Figure S1). However, there is no significant trough between the Spring and Summer peaks. Also, the Summer peak, rather than the Winter peak, represented the dominant outbreak of the 2022–2023 seasonal year. This is, however, deceitful and likely secondary to the significantly decreased reporting of daily cases by state and local governments in the Winter 2022–2023 season (20), as we discuss in the discussion section below.

**Table 1:** Date and daily confirmed case count at each of the local maxima (peaks) for the 2020–2021 and 2021–2022 seasonal years.

Season	2020–2021			2021–2022		
	Peak date	Cases (spline)^a^	Cases (raw)^b^	Peak date	Cases (spline)^a^	Cases (raw)^b^
Spring	4/21/2020	29,236	37,162	4/12/2021	67,716	105,154
Summer	7/22/2020	61,325	77,048	8/29/2021	171,056	314,495
Fall	–	–	–	–	–	–
Winter	12/25/2020	220,836	333,900	1/14/2022	621,411	1,408,577

Dates are written in M/D/Y format. Peak date is obtained as the local maximum at each seasonal peak of the cubic spline fitted to the raw data of Figure 1A.

a Estimated case counts at each peak date obtained from the cubic spline fit to the raw data.

b Maximum raw case counts at ±10 days from the peak date (as peak dates from the smoothed cubic spline do not exactly correlate with the maximum daily case count from the raw data at each peak).

### Characterization of the periodic components in incidence

We characterized the periodic components in COVID-19 incidence using spectral analyses. Scaled periodogram values over all Fourier frequencies were computed as described in Eq. 3 and are plotted in Figure 2. The periodogram shows four dominant peaks, whose values and frequencies are shown in Table 2. The largest peak has a frequency of 0.00273 days^−1^ and period of 366 days, representing a sinusoid with a single peak per year. The next largest periodogram value is at frequency of 0.00683 days^−1^ and period of 146.4 days. The final two significant peaks have periods of 183 and 122 days and frequencies of 0.00546 days^−1^ and 0.00820 days^−1^, respectively. The peak representing the period of 91.5 days had a scaled periodogram value of 0.085, which was below the 0.1 cutoff value for our study.

**Table 2:** Period, frequency, periodogram value (Eq. 3), and scaled periodogram value (Eq. 4) of each of the dominant peaks found in the periodogram in Figure 3.

Dominant period (1/*ω*)	Frequency (*ω*)	Periodogram value	Scaled periodogram value
366 days/cycle	0.00273 days^−1^	154.8	0.846
146.4 days/cycle	0.00683 days^−1^	51.1	0.279
183 days/cycle	0.00546 days^−1^	34.4	0.188
122 days/cycle	0.00820 days^−1^	28.6	0.157
91.5 days/cycle	0.0109 days^−1^	15.6	0.085

A scaled periodogram value of 0.1 was set as the threshold for significance.

In Figure 2D, we present a smoothed periodogram in which a Daniel kernel is applied to the periodogram values, which averages the values as shown:


(8) $$s_{k}=\frac{1}{3}u_{k-1}+\frac{1}{3}u_{k}+\frac{1}{3}u_{k+1}$$

Where *s_k_* is the smoothed periodogram value and *u_k_* is the unsmoothed value, for all *k* = 2, …, 731. The smoothed periodogram shows the two largest peaks with periods 366 and 146.4 days remain significant whereas the other peaks are smoothed out. This is evidence that the other peaks surrounding the 146.4 days peak in the raw periodogram were likely a result of noise or imperfect sinusoidal activity within the data. The effect of this noise is also evidenced by the appearance of isolated clusters of small peaks at frequencies ~0.14, ~0.28, and ~0.43 in Figure 2A. These peaks, called harmonics, are the result of non-sinusoidal behavior in the data. On further inspection, the harmonics occur at multiples of the frequency 105/732, and can be represented by $\omega_{h,k}=k\omega_{h}$ , where *ω_h_* = 105/732 and *k* = 1, 2, …, 7. When calculating periodogram values using points from the cubic spline in Figure 1B, the harmonics disappear while the major peaks remain (Figure 2B) due to the fact that the smoothed cubic spline eliminates the noise in the data. For the sake of completeness, we also calculated periodogram values for the 2022–2023 seasonal year alone. In order to overcome the significant noisiness in the data, we used the 7 days moving average around each datapoint (as seen in Supplementary Figure S1B) when calculating the periodogram values. Three significant periodogram peaks were found at the following three adjacent frequencies: 0.00282, 0.00563, and 0.00845 days^−1^ in order from the greatest to the least periodogram value (Supplementary Figure S2). The periods represented by these frequencies are: 355 days, 177.5 days, and 118.3 days, respectively.

### Plotting the theoretical sinusoids

Using the frequencies of the dominant periods in the data (Table 2), we constructed the theoretical sinusoids to be fit to the data using Eqs. 5–7 above. Figure 3 shows the best fit sinusoid for each of the four significant periods in order of largest periodogram value, as well as the Pearson’s correlation coefficient (𝝆) of that sinusoid with the data. Cross-correlation functions plotted for each of these theoretical sinusoids confirm that each sinusoid shown is best fit to the data (Supplementary Figure S3). All best fit sinusoids had a strong positive correlation with the data.

In Figure 3A, the sinusoid with period 366 days predicts the general increase in cases, when overlooking the local minima, as the year progresses towards the annual dominant peak in the Winter season followed by a drop in cases toward an annual minimum in the late Spring/early Summer. This explains the very strong positive correlation of this sinusoid with the incidence data (𝝆 = 0.744). Figures 3B–D show the sinusoids that capture the multiple peaks each year. The sinusoid with period of 146.4 days (Figure 3B) had a strong positive correlation (𝝆 = 0.413) with the data. Although it does not fit well to the initial, smallest peak in the Spring of 2020, it fit very well to each of the peaks and troughs of the 2021–2022 seasonal year. The sinusoid with period 183 days did not align well visually with several of the peaks in the data, though it had a good correlation (𝝆 = 0.346). Lastly, the sinusoid with period of 122 days aligned well with most of the peaks; however, the width of its peaks was much smaller than most of the peaks in the data, resulting in a lower correlation (𝝆 = 0.295).

Upon separating the data into two separate years, the sinusoid with period of 146.4 days aligns very well with each of the 3 annual COVID-19 peaks and fits the width of each peak most accurately (Figure 4). In the 2020–2021 year (Figure 4A), there was a slight improvement in the correlation of the best fit sinusoid (𝝆 = 0.431), but it did not fit the initial 2020 Spring peak well. The CCF for the sinusoids in Figure 4A (Supplementary Figure S4) shows that a positive lag (rightward shift) of 11 days results in the greatest correlation (𝝆 = 0.500) of the theoretical sinusoid with the data. This significant increase in the correlation of the lagged sinusoid is due to the truncation of the initial 11 data points of the data where there was a misalignment with the first peak in the Spring of 2020, resulting in a sinusoid that matches each of the peaks and troughs much more accurately. In Figure 4B, the sinusoid with period 146.4 days has a very good fit to the 2021–2022 data with peaks and troughs matching within ±10 days (except the first peak where the difference was 20 days), resulting in a very strong positive correlation (𝝆 = 0.730).

## Discussion

Definition and quantitative characterization of COVID-19 seasonality are not only critical to the understanding of the COVID-19 pandemic, but also needed for planning economics and public health policies. The current work describes an in-depth statistical study of the waves in the epidemic spreading of COVID-19 in two full calendar years, from March 19, 2020, to March 20, 2022, in the contiguous United States. We did not include the data of COVID-19 incidences collected from 2022 to 2023 because the testing and case reliability were significantly decreased in the final year of case tracking.

Our analyses uncovered an obvious periodicity in COVID-19 incidences and revealed four dominant periods of oscillation in the US over the 2 years of data analyzed (Figures 3, 4). Specifically, the two most dominant periods of oscillation were 366 days and 146.4 days. The period of 366 days indicates that there is a single dominant COVID-19 outbreak that occurs approximately once every year, which correlates with the outbreak seen in the early/mid-Winter months. However, it is obvious by just mere observation that there exists more than just a single annual peak in the data. Rather, this periodogram peak is likely the result of the large proportion of cases lying in the Winter months, thus skewing the correlation in favor of this dominant yearly peak. The period of 146.4 days indicates approximately 3 peaks in incidence per year that aligns the 3 annual outbreaks: the dominant Winter peak mentioned above and two smaller peaks during mid-Spring and mid-Summer. This sinusoid appears to characterize the data most accurately as it fits very well to each of the 3 yearly peaks in the data (Figures 3, 4). The periodogram also indicated two smaller peaks with sinusoidal periods of 183 days and 122 days. These peaks likely result from imperfect sinusoidal activities within the data surrounding the periodogram peak attributed to the sinusoid with period of 146.4 days, and are not accurate representations of the data (Figures 3C,D). Though not included in the main analysis, we also showed that the dominant periods of oscillation in the 2022–2023 data were 355 days, 177.5 days, and 118.3 days. This emulates the oscillatory periods in the 2020–2022 data, as the dominant period is that of 355 days due to the dominating yearly peak in incidence, and the periods of 177.5 and 118.3 days are similar to the 146.4 days period of the 2020–2022 data, again indicating that there exists a tri-annual outbreak pattern, as can be seen in Supplementary Figure S1B.

The results we achieved suggest that a single seasonal COVID-19 pattern that repeats over a 1 year fixed period cannot be observed in the US (Figure 1). Instead, our analysis identified several outbreaks each year, which appear to occur with a fixed frequency. Unlike seasonal patterns seen in viruses such as the Influenza virus (Supplementary Table S1), where annual outbreaks are closely related to environmental factors such as temperature and humidity (21), climate does not appear to play a major role in determining COVID-19 outbreaks. While there is a clear and regular seasonal pattern in the outbreaks, they occur across multiple seasons between which climate varies significantly. Thus, although climatological factors may be involved in the occurrence of COVID-19 outbreaks, it appears that there exist more dominant factors that determine the dynamics of the SARS-COV-2 virus which remain to be fully elucidated.

Seasonal patterns in COVID-19 incidences have also been found in other countries. A recent Fourier spectral analysis of the SARS-CoV-2 cases across 30 countries revealed the recurrence of at least one COVID-19 wave, often two or more, repeating over a variable period, in the range of 3 to 9 months (7). Indeed, the studies on the COVID-19 waves across the world showed modest variation in the seasonality of COVID-19 incidences, likely affected by environmental factors, travel restriction or lockdown policies, and vaccination campaign (8, 22–24). For example, in Japan and United Kingdom, infection waves of about 170 days were observed during the vaccination campaign (23). The studies on COVID-19 in Nigeria and Uganda showed interesting seasonality results, but only 2 peaks per year in these countries (2). The data showed that more cases of COVID-19 are expected in the first (January–March) and third (July–September) quarters of a year in Nigeria and Senegal whereas in DRC and Uganda, more cases of COVID-19 may likely be reported in the second (April–June) and fourth (October–December) quarters (Supplementary Table S1).

By identifying the predictable seasonality of COVID-19 outbreaks, our findings provide important information for public health preventative efforts to control future outbreaks. Supplies can be distributed, vaccination efforts can be focused and timed, and in-hospital/community preparations can be made in anticipation of predictable outbreaks. For even the most efficacious vaccines, vaccine effectiveness (VE) has been found to peak 1–2 months following vaccine administration then decline monotonically thereafter (10). Specifically, the peak VE for mRNA-based vaccines appears to be approximately 2 months following administration. Thus, based on our results for the 2020–2022 seasons, it would be beneficial to administer mRNA-based booster vaccinations to patients in the United States approximately 2 months before the expected Winter case spike, e.g., in mid-October/early November from the current data. At first glance it may seem as though the Winter season of the 2022–2023 dataset was the smallest of the 3 incidence peaks in that year (Supplementary Figure S1B). However, this is likely untrue and likely secondary to decreased daily case reporting, as daily COVID-19 hospitalizations during the 2022–2023 seasonal year were highest during the Winter season (20). One could also argue that a bi-annual booster vaccine schedule may be beneficial for high-risk and immunocompromised patients to ensure year-round coverage due to the multiple yearly peaks and the significant loss in VE 7 months after administration (from 95.9% VE at 2 months to 80.3% VE at 7 months post-administration). In addition to instilling booster vaccine schedules, COVID-19 testing should be encouraged in symptomatic and high-risk patients and public awareness of the benefits of hand washing, face-masks, and social distancing should be stressed during predicted incidence peaks in mid-Spring, -Summer, and -Winter (11–13). Finally, as coronaviruses have repeatedly caused multiple deadly epidemics in the past, such as the severe acute respiratory syndrome (SARS-CoV-1) and middle eastern respiratory syndrome (MERS), seasonality studies are crucial to understanding the patterns of coronavirus infections. Thus, though the patterns we see in the initial years of the COVID-19 pandemic may change, these studies provide pertinent information on the initial dynamics of the viruses and will be critical in guiding anticipatory public health efforts to guard against case surges in future coronavirus epidemics/pandemics.

The strength of this study is that we successfully identified and characterized a tri-annual peak pattern in COVID-19 incidences in the United States. These proposed incidence waves showed excellent goodness of fit to the raw data. Thus, our results are rigorous to vaccination campaigns and other public health measures which greatly affected results in other studies on disease periodicity (23, 25). Furthermore, we are able to use these predictable incidence waves to make public health recommendations to guide the timing of vaccinations and non-pharmacological infection control measures, as discussed above. Additionally, our study is unique in that it utilizes virtually all available daily United States COVID-19 incidence data across three separate seasonal years. This is the most reliable data that will likely be available for this pandemic, as daily incidence reporting has halted due to the declining availability of data at both state and local levels (20). Currently, the most reliable estimations of disease incidence are weekly data reports of COVID-19 hospitalization and test-positivity rates posted by the CDC (26). This may affect the accuracy of conclusions in future studies as hospitalization and test-positivity data are plagued with biases due to their dependence on disease severity, which changes drastically with viral strains and vaccination (27), testing procedures and the decrease in result reporting secondary to increased home testing (28), and selection for symptomatic individuals. Furthermore, regardless of future data, our study describes the early dynamics of the COVID-19 disease at the most important portion of the pandemic in both its early stages and its climax.

Our study is not without limitations, however. The data available to analyze was limited by the fact that the pandemic has only existed for three full seasonal years, the final year of which consisted of very noisy data that was difficult to draw accurate conclusions from. Thus, it is very possible that disease dynamics may change over coming years, and the conclusions from this study must be re-validated in the future as more years of data become available. Furthermore, our conclusions may be limited by the fact that we did not adjust the data for emergence of new viral variants, vaccination rates, lockdown orders, social distancing, mask mandates, and public holidays. However, a main aim of our study was to use raw case data to identify disease dynamics as it co-existed with these factors. We showed that the patterns seen remained consistent regardless of whether these factors existed or not. Thus, these patterns can be considered inherent characteristics of the disease in its early stages, independent of initial public health efforts.

Lastly, it should be noted that, although the outbreaks can be predicted by the measures we described here, our study cannot be used to predict the amplitudes of the incidences in each outbreak. The amplitude of a COVID-19 outbreak is complex and depends on a multitude of variables that are yet to be fully elucidated, including the emergence of new SARS-CoV-2 strains, social distancing efforts, vaccination, and environmental factors, etc. This is evidenced by the lack of a dominating peak in the Winter of the 2022–2023 seasonal year (Supplementary Figure S1) whereas this peak was dominant in the 2020–2021 and 2021–2022 seasonal years.

## Future directions

This study lays the foundations of characterizing the seasonality of COVID-19 incidence in the United States; next, it will be essential to identify the cause of this regular periodicity. As mentioned above, these incidence waves were impervious to vaccination campaigns, did not directly correlate with public holidays, and did not seem to be correlated with any environmental factors. Rather, research on inherent properties of the virus, such as studies on the association of variant emergence, waning host immunity, and viral transmission and stability with these regular periods, should be conducted to uncover potential mechanisms behind its seasonality. Additionally, the amplitudes of peak incidences varied greatly with each outbreak. Though environmental factors have been found to be significantly associated with COVID-19 incidence (29), the variation in amplitudes of peaks appears to be multifactorial and likely involves a combination of environmental, public health, immunological, and viral factors. Further studies will be needed to elucidate the interplay between these factors which resulted in the peak amplitude variation which we saw in this study. Finally, as we implement the highlighted public health interventions to combat these predicted surges, it will be necessary to revisit these studies with future data to assess whether the seasonality of COVID-19 evolves over time.

## Data availability statement

The original contributions presented in the study are included in the article/Supplementary material, further inquiries can be directed to the corresponding authors.

## Ethics statement

Ethical approval was not required for the study involving humans in accordance with the local legislation and institutional requirements. Written informed consent to participate in this study was not required from the participants or the participants’ legal guardians/next of kin in accordance with the national legislation and the institutional requirements.

## Author contributions

ES: Conceptualization, Investigation, Validation, Writing – original draft, Data curation, Methodology. AS: Validation, Writing – review & editing. KZ: Conceptualization, Investigation, Funding acquisition, Project administration, Resources, Supervision, Writing – original draft, Validation, Writing – review & editing.

## Footnotes

^1^ https://coronavirus.1Point3Acres.com
