# Supplementary material for: Seasonality of COVID-19 incidence in the United States
Source: Front Public Health. 2023 Dec 5;11:1298593. doi: 10.3389/fpubh.2023.1298593 (PMC10728821; doi:10.3389/fpubh.2023.1298593)
Supplement: Supplementary file 1 [file Data_Sheet_1.PDF]

Supplemental Information

Supplemental Table 1. Comparison of Seasonality of Viral Pandemics

Virus	Study	Country	Start Season	Season Peak	End Season	Peak frequency
SARS-COV-2	Current study; Adebowale et al. (1)	A) USA<br>B) Nigeria and Senegal<br>C) Democratic Republic of Congo and Uganda	–	A)<br>1) Apr<br>2) July - Aug<br>3) Dec - Jan<br>B)<br>1) Jan - Mar<br>2) July - Sept<br>C)<br>1) Apr - June<br>2) Oct - Dec	–	A) 3 annual peaks<br>B) 2 annual peaks<br>C) 2 annual peaks
Influenza	Tamerius et al. (2)	A) USA<br>B) Brazil<br>C) Singapore<br>D) Australia	A) Nov<br>B) Jan<br>C) –<br>D) June	A) Dec - Jan<br>B) Mar - Apr<br>C)<br>1) June - July<br>2) Dec - Jan<br>D) Aug - Sept	A) Apr - May<br>B) July<br>C) –<br>D) Oct	A) 1 annual peak<br>B) 1 annual peak<br>C) 2 annual peaks<br>D) 1 annual peak
Rhinovirus	Monto A. S.(3); Lau et al (4).	USA	Sep	Oct	Dec	1 annual peak
Enterovirus	Pons-Salort et al.(5); Moore M. (6)	USA	Jun	Aug - Sep	Oct	1 annual peak
HPIV1*	Abedi et al. (7)	USA	May - July	Sep - Oct	Dec - Jan	1 biennial peak
HPIV3*	Abedi et al. (7)	USA	Mar	Apr - July	Aug	1 annual peak
RSV	Obando-Pacheco et al. (8)	A) USA<br>B) Brazil<br>C) China	A) Nov<br>B) March<br>C) Nov	A) Jan<br>B) Apr - May<br>C) Dec - Feb	A) Apr<br>B) July<br>C) Apr	1 annual peak

Table note:
*HPIV1 = Human parainfluenza 1; HPIV3 = Human parainfluenza 3

SARS-COV-1 was quickly contained and not enough data exists to determine seasonality. MERS-CoV (Middle Eastern Respiratory Syndrome Coronavirus) did not demonstrate blatant seasonality, however some epidemiological studies found that cases tended to peak in June.

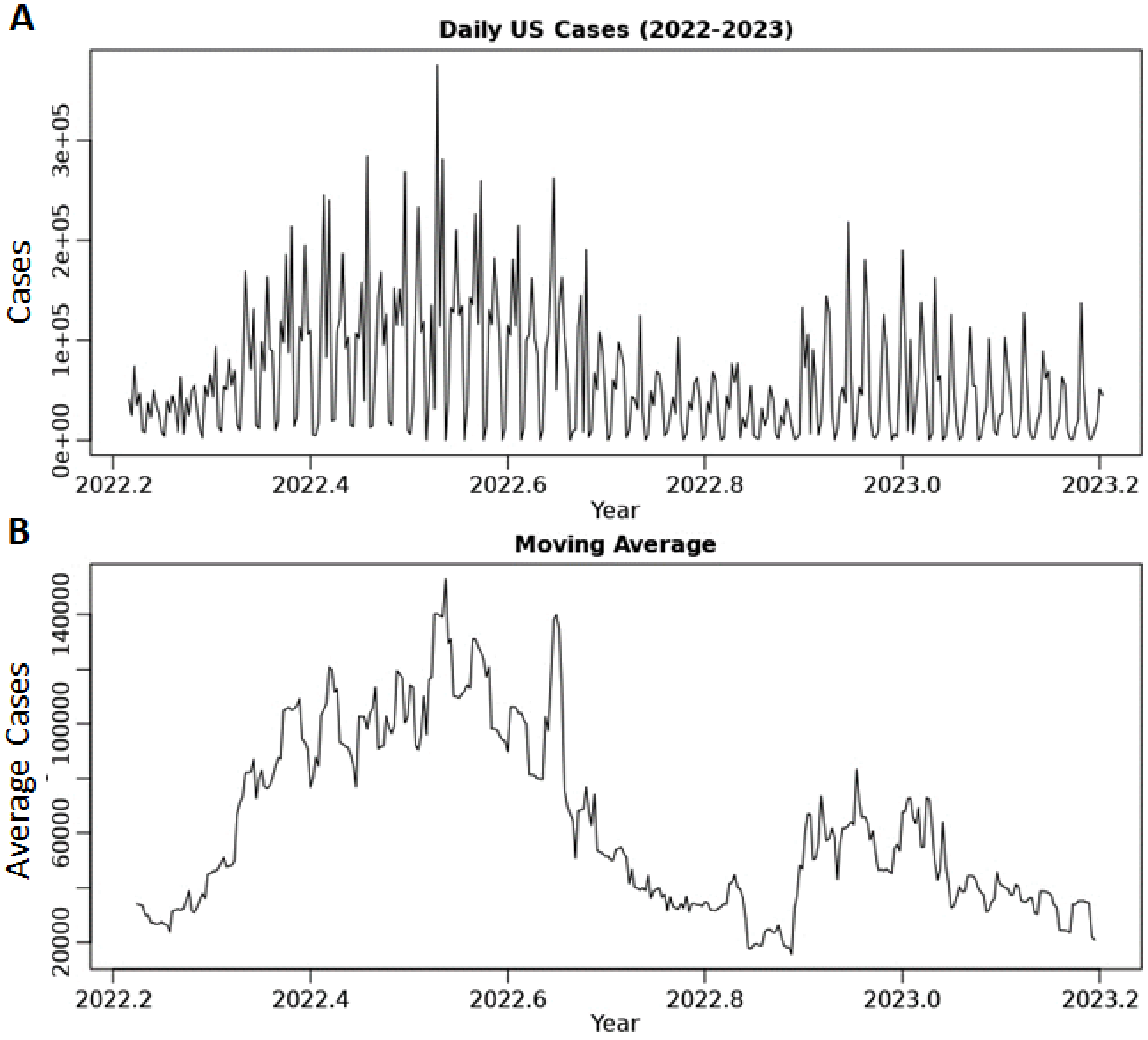

Supplemental Figure 1. Daily COVID-19 incidences in the USA over the 2022-2023 seasonal year. **A** shows the raw data. **B** shows the 7-day moving average (centered on the time-point in question).

A

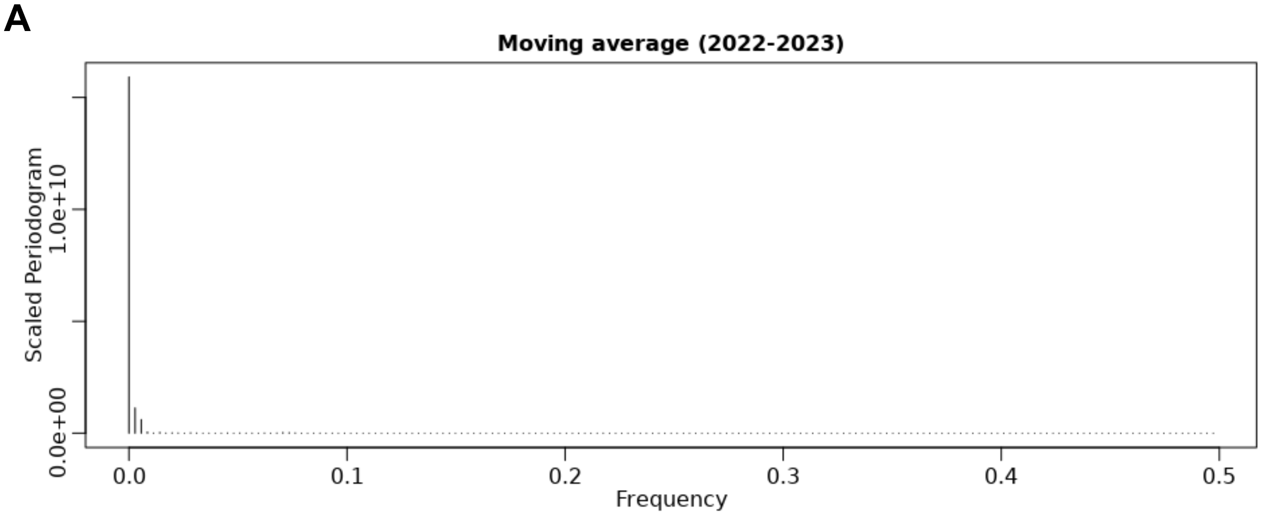

B

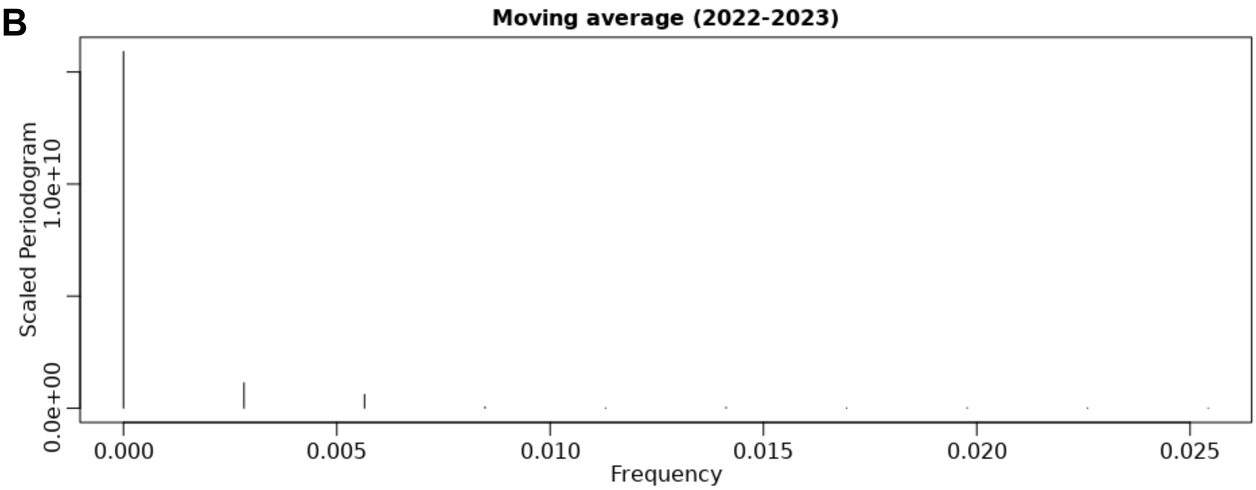

Supplemental Figure 2. Periodogram of COVID-19 incidence for the 2022-2023 seasonal year. The 7-day moving average around each datapoint in the raw data was used to calculate the periodogram values. n = 355 days, and frequency represents number of cycles per 355 days. **A** shows all periodogram values. **B** shows a zoomed in view showing the 3 major periodogram peaks at frequencies: 0.002816901, 0.005633803, and 0.008450704 $days^{-1}$. The periods represented by these frequencies are: 355 days, 177.5 days, and 118.3 days, respectively.

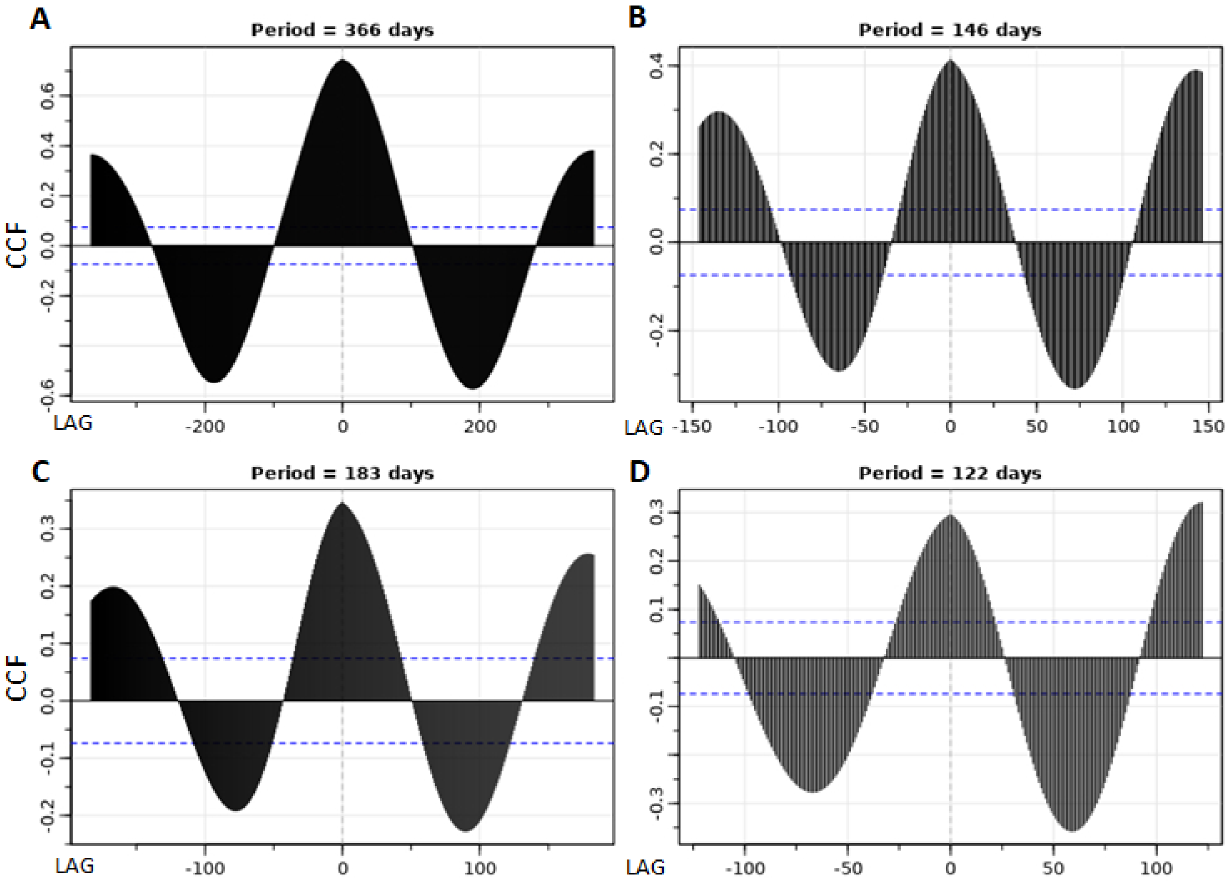

Supplemental Figure 3. Cross-correlation functions for each of the plots in Figure 3, where the theoretical sinusoid is lagged with respect to the cubic spline fitted to the data. X-axis is the positive and negative lag (in days) for approximately one full period of the sinusoid. Y-axis is the correlation of the sinusoid with the cubic spline. The dashed blue lines indicate significant correlation; however, this significance level only applies when the data is pre-whitened (i.e., one of the time-series is noise), thus these can be disregarded.

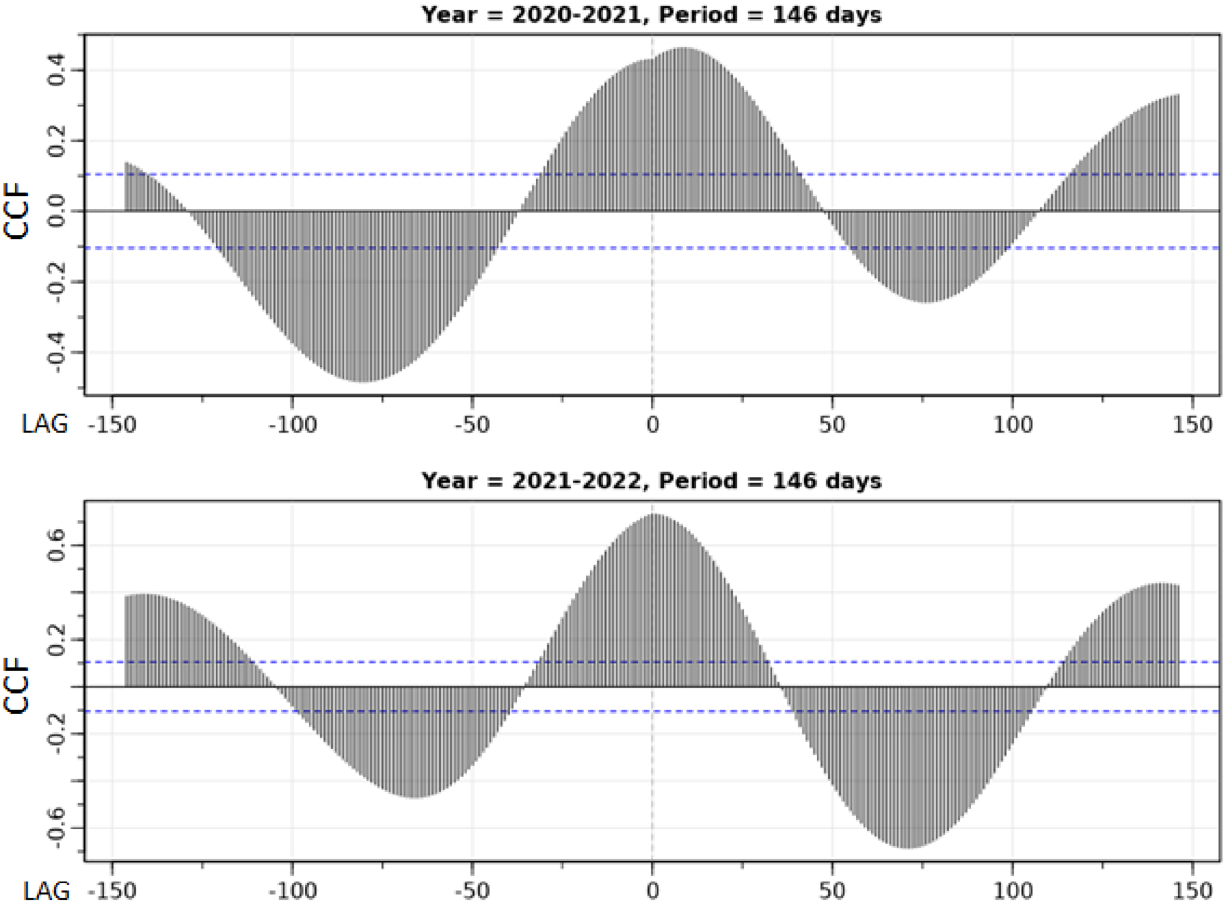

Supplemental Figure 4. Cross-correlation functions for each of the plots in Figure 4, where the theoretical sinusoid is lagged with respect to the cubic spline fitted to the data. X-axis is the positive and negative lag (in days) for approximately one full period of the sinusoid. Y-axis is the correlation of the sinusoid with the cubic spline. The dashed blue lines indicate significant correlation; however, this significance level only applies when the data is pre-whitened (i.e., one of the time-series is noise), thus these can be disregarded.

References:

1. Adebowale AS, Afolabi RF, Bello S, Salawu MM, Bamgboye EA, Adeoye I, et al. Spread and seasonality of COVID-19 pandemic confirmed cases in sub-Saharan Africa: experience from Democratic Republic of Congo, Nigeria, Senegal, and Uganda. *BMC Infect Dis.* 2023;23(1):187.

2. Tamerius J, Nelson MI, Zhou SZ, Viboud C, Miller MA, and Alonso WJ. Global influenza seasonality: reconciling patterns across temperate and tropical regions. *Environ Health Perspect.* 2011;119(4):439-45.
3. Monto AS. The seasonality of rhinovirus infections and its implications for clinical recognition. *Clin Ther.* 2002;24(12):1987-97.
4. Lau SK, Yip CC, Woo PC, and Yuen KY. Human rhinovirus C: a newly discovered human rhinovirus species. *Emerg Health Threats J.* 2010;3:e2.
5. Pons-Salort M, Oberste MS, Pallansch MA, Abedi GR, Takahashi S, Grenfell BT, et al. The seasonality of nonpolio enteroviruses in the United States: Patterns and drivers. *Proc Natl Acad Sci U S A.* 2018;115(12):3078-83.
6. Moore M. Centers for Disease Control. Enteroviral disease in the United States, 1970-1979. *J Infect Dis.* 1982;146(1):103-8.
7. Abedi GR, Prill MM, Langley GE, Wikswo ME, Weinberg GA, Curns AT, et al. Estimates of Parainfluenza Virus-Associated Hospitalizations and Cost Among Children Aged Less Than 5 Years in the United States, 1998-2010. *J Pediatric Infect Dis Soc.* 2016;5(1):7-13.
8. Obando-Pacheco P, Justicia-Grande AJ, Rivero-Calle I, Rodriguez-Tenreiro C, Sly P, Ramilo O, et al. Respiratory Syncytial Virus Seasonality: A Global Overview. *J Infect Dis.* 2018;217(9):1356-64.
